# Photoreceptor Processing Speed and Input Resistance Changes during Light Adaptation Correlate with Spectral Class in the Bumblebee, *Bombus impatiens*

**DOI:** 10.1371/journal.pone.0025989

**Published:** 2011-10-27

**Authors:** Peter Skorupski, Lars Chittka

**Affiliations:** Biological and Experimental Psychology Group, School of Biological and Chemical Sciences, Queen Mary University of London, London, United Kingdom; Lund University, Sweden

## Abstract

Colour vision depends on comparison of signals from photoreceptors with different spectral sensitivities. However, response properties of photoreceptor cells may differ in ways other than spectral tuning. In insects, for example, broadband photoreceptors, with a major sensitivity peak in the green region of the spectrum (>500 nm), drive fast visual processes, which are largely blind to chromatic signals from more narrowly-tuned photoreceptors with peak sensitivities in the blue and UV regions of the spectrum. In addition, electrophysiological properties of the photoreceptor membrane may result in differences in response dynamics of photoreceptors of similar spectral class between species, and different spectral classes within a species. We used intracellular electrophysiological techniques to investigate response dynamics of the three spectral classes of photoreceptor underlying trichromatic colour vision in the bumblebee, *Bombus impatiens*, and we compare these with previously published data from a related species, *Bombus terrestris*. In both species, we found significantly faster responses in green, compared with blue- or UV-sensitive photoreceptors, although all 3 photoreceptor types are slower in *B. impatiens* than in *B. terrestris*. Integration times for light-adapted *B. impatiens* photoreceptors (estimated from impulse response half-width) were 11.3±1.6 ms for green photoreceptors compared with 18.6±4.4 ms and 15.6±4.4 for blue and UV, respectively. We also measured photoreceptor input resistance in dark- and light-adapted conditions. All photoreceptors showed a decrease in input resistance during light adaptation, but this decrease was considerably larger (declining to about 22% of the dark value) in green photoreceptors, compared to blue and UV (41% and 49%, respectively). Our results suggest that the conductances associated with light adaptation are largest in green photoreceptors, contributing to their greater temporal processing speed. We suggest that the faster temporal processing of green photoreceptors is related to their role in driving fast achromatic visual processes.

## Introduction

Trichromatic colour vision depends on three classes of photoreceptor with different spectral sensitivities across the visible spectrum. In the trichromatic insects, there is relatively little variation in spectral tuning [1], [2], [3] but photoreceptors may differ in other respects than spectral sensitivity, for example in temporal processing [4]. In fact there is a well-established link between visual ecology and species-typical receptor processing speed for achromatic vision [4], [5]. Classic studies in dipterans have demonstrated that fast photoreceptor responses are confined to highly active, fast-flying species, while slower, less manoeuvrable species have correspondingly slower photoreceptors [5]. Since fast membrane responses require large membrane conductances (to reduce time constants), which in turn incurs a substantial metabolic cost (increased ion pumping to maintain concentration gradients), this suggests that any overcapacity in the performance of the neuronal membrane is penalized [6]. The preceding studies are based on between-species comparisons of equivalent receptor types, namely the longer-wave sensitive (green) receptor in hymenopterans, the broadband, double-peaked R1-6 receptor in dipterans [5], and spectrally unidentified (but presumably longer wave, broadband) receptors in several other orders [7]. Within a species, however, receptors of different spectral sensitivity can also display electrophysiological differences [8], which are reflected in differences in temporal processing [9].

We have recently compared temporal processing between spectral classes within a species, the bumblebee *Bombus terrestris* [10]. In a manner analogous to parallel visual processing in humans, bees process chromatic and achromatic signals via separate channels [11], [12], [13], [14]. Many visual functions, including perception of depth and motion can be driven solely by green photoreceptor contrast, and are therefore achromatic. The main function of blue and UV photoreceptors appears to be to add a chromaticity signal for colour vision (although UV photoreceptors are also involved in polarization vision, and may also drive certain stereotypical, wavelength-dependent behaviours [15]). We found that green photoreceptors, under light-adapted conditions, generated significantly faster responses than blue or UV photoreceptors [10]. Since fast temporal processing is metabolically expensive [16] it may make sense to economize on speed of chromatic processing. In the present study we investigate electrophysiological properties of different spectral classes of photoreceptor in the bumblebee, *Bombus impatiens* (a common model system in visual ecology, and importantly, recent studies have begun to provide neuroanatomical and physiological data on central visual processing in this species [13], [17], [18]). We show that, as in *Bombus terrestris*, green photoreceptors (driving fast achromatic visual processes) generate faster responses than blue or UV photoreceptors (which are used in chromatic vision). To the extent that these differences are due to differential tuning of the membrane frequency response by different sets of voltage-gated conductances, one would expect a given level of light-induced membrane depolarization to be associated with a larger conductance increase in green photoreceptors than in blue and UV photoreceptors. Here we show that the decrease in input resistance (reflecting increased membrane conductance) during light adaptation is indeed greatest in green photoreceptors.

## Materials and Methods

### Preparation and recording

Worker bumblebees used in these experiments were obtained from commercially available colonies of *Bombus impatiens* (supplied by Biobest Bees, Leamington, Canada). These same animals were used in a previously reported study of photoreceptor spectral sensitivity in *Bombus impatiens* [19]. The preparation, and recording and stimulating techniques, were the same as described previously in detail for *Bombus terrestris* [2], [10], and are described more briefly here.

Photoreceptor impulse responses were measured using high-intensity LEDs to generate flashes of 0.05–1.0 ms in duration. Responses to 100–400 such pulses, delivered at a repetition rate of 1–2.5 Hz were then averaged. The peak wavelengths of the LEDs were in the near-UV (360 nm, 15 nm half-width, FoxUV, DComponents, VT, USA) or blue (470 nm, 22 nm half-width) region of the spectrum. Peak spectral sensitivity of *Bombus impatiens* blue and green photoreceptors are at 424 nm and 539 nm; at 470 nm the relative sensitivities are about 0.25 and 0.5, respectively [19]. The peak spectral sensitivity of the UV receptor is 348 nm, and the relative sensitivity at 360 nm is 0.86.

We measured light intensity in relative log units with respect to source attenuation, as in our previous study [10], rather than attempting to calibrate the effective quantum catch of individual photoreceptors. Although we observed photoreceptor noise indicative of individual and summed quantum events, single bumps could not be identified reliably following the 10 min or more of dark adaptation used here, suggesting that bumblebee photoreceptors require long periods to become deeply dark-adapted. In support of this we noted increases in baseline noise in the dark, of several seconds duration, following 10 ms flashes of low to moderate intensity (attenuated so as to limit peak response amplitude to <10 mV).

Input resistance measurements were made using an Axoclamp 2B (Molecular Devices, CA, USA) in discontinuous current clamp mode, with a switching frequency between 1.7 and 2.5 kHz. Settling of the voltage responses was always monitored on a separate dedicated oscilloscope. Resting membrane potential was determined on withdrawal of the microelectrode following recording, where possible. Resting potential values were accepted if withdrawal of the electrode resulted in (1) an immediate positive shift of potential >50 mV, and (2) monophasic negative-going responses to brief flashes (the ERG).

### Measurement and analysis

Individual sensitivity functions (*V*/log *I* functions) were obtained for individual photoreceptors as described previously [19].

Impulse responses were fitted with the lognormal function as described previously [7], [10].
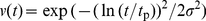
(1)In this equation *t*_p_ is the time to peak, and *σ* is a shape parameter. Smaller *σ* values produce more symmetric responses, and larger values more positively-skewed and broader responses. For a given *t*_p_, therefore, increasing *σ* increases response duration.

Where mean values are given in the text, errors are quoted as one standard deviation.

## Results

**Figure 1:**
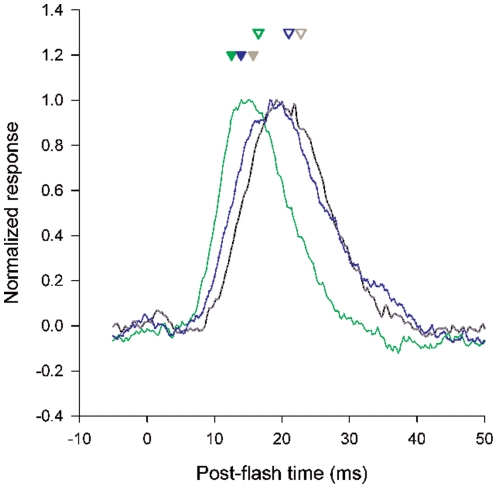
Impulse responses in green photoreceptors peak more rapidly and are completed earlier than those of blue or UV photoreceptors. Averaged impulse responses (300–500 sweeps per average) recorded from three photoreceptors from the same bee, normalized and superimposed for comparison. Actual response amplitudes were 1.2 mV, 1.2 mV and 1.3 mV for the green-, blue- and UV-sensitive photoreceptors, respectively. Impulse responses were recorded following 60 s adaptation to steady light at 470 nm (−0.75 log units intensity; green, blue photoreceptors) or 360 nm (−1.87 log units; UV photoreceptor). The mean depolarization of the resting membrane potential during light adaptation was 22.3 mV (green photoreceptors), 22.7 mV (blue) and 23.6 mV (UV). For comparison, mean values of the impulse response time-to-peak (*t*_p_ ) are indicated for green, blue and UV photoreceptors in *Bombus terrestris* [10] (solid arrows, green, blue and grey, respectively), along with corresponding means for all photoreceptors in *Bombus impatiens* measured in this study (open arrows).

Stable intracellular recordings of greater than 60 minutes duration were obtained from 20 *Bombus impatiens* photoreceptors, from three workers. Measurement of impulse responses and negative contrast responses yielded similar results to those reported previously in *Bombus terrestris* [10]. Following initial spectral characterization [19] of a cell as either a UV (n = 4), blue (n = 6), or green photoreceptor (n = 10) the preparation was usually left to dark-adapt for 10 minutes. Impulse responses to brief pulses of light, adjusted in duration between 0.1 and 1 ms in order to generate averaged impulse responses of 1.0±0.5 mV were somewhat variable in duration when delivered in dark conditions. However, such responses were well-fitted with the log-normal function. The time-to-peak, *t*_p_, ranged from 31–89 ms, with no significant difference between receptor spectral classes (green: 40.4±5.1 ms; blue: 46.3±3.7; UV: 56.4±3.3). This variability may have been due to incomplete dark adaptation; prior to recording photoreceptors were exposed to brief but moderately intense flashes in order to determine spectral type and *V*/log *I* function. It seems likely that long periods of darkness (30–60 minutes) would be required for the cells to become ‘deeply dark adapted’ [5]. Following light adaptation all photoreceptors generated faster impulse responses, although to a variable extent (Fig. 1). Low or moderate intensities of adapting light (generating steady state depolarization of <10 mV) generated moderate decreases in *t*_p_. The maximum decrease in *t*_p_ appeared complete with moderately intense adapting lights that depolarized the cells by more than about 10 mV from the dark resting membrane potential; further increases in the adapting light intensity (generating a steady state depolarization up to 30 mV depolarized from rest) did not lead to further decreases in *t*_p_; in fact in some cells impulse responses generated at the highest adapting intensities showed a slight *increase* in *t*_p_ compared to moderate intensities. Since the probability of maintaining stable, long term intracellular recording seemed to decrease following prolonged adaptation to the highest intensities used here, in most experiments we adjusted the intensity (depending on receptor spectral class) to generate steady state depolarizations of 10–20 mV above rest. With reference to the *V*/log *I* functions for the same photoreceptors, the initial peak depolarization in response to onset of the adapting light typically corresponded to *V*/*V*_max_ values of about 0.5–0.8, and the steady-state light-adapted depolarization to about 0.3–0.5 [19].

**Figure 2:**
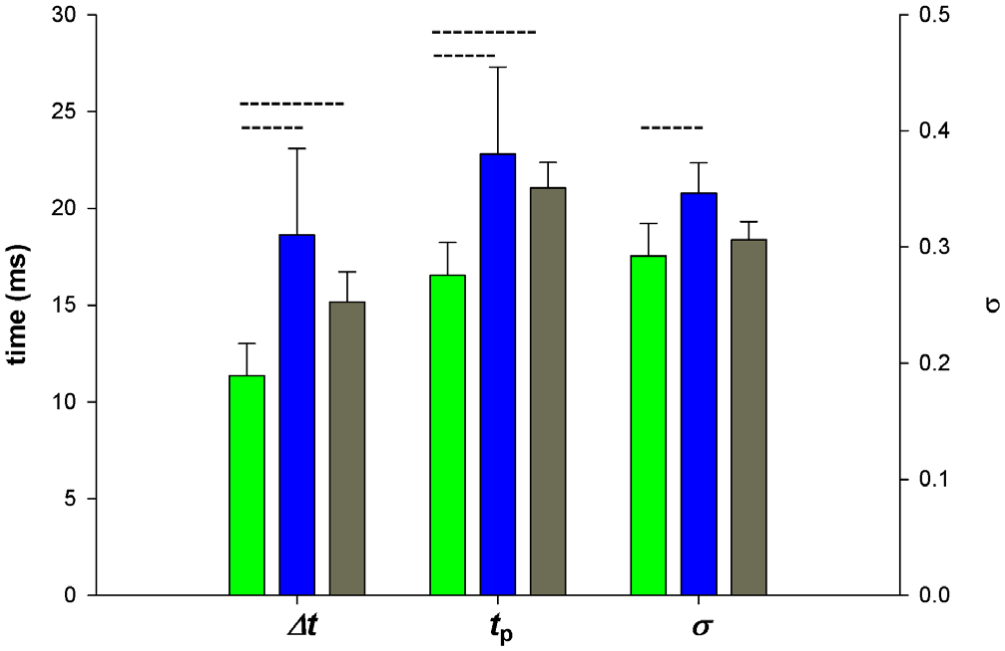
Mean parameters of light-adapted impulse responses from all three photoreceptor spectral classes. Green, blue and grey bars denote green, blue and UV photoreceptor respectively. Left columns: *Δt* (half-width) in ms; middle columns: *t*_p_ in ms; right columns: *σ*, plotted on the dimensionless y-axis on the right. Mean light-adapted membrane potential was 22.8±3.5 mV (green), 23.1±5.7 (blue), 21.8±3.6 (UV).

Faster photoreceptor impulse responses were due to both a decrease in *t*_p_ and a decrease in the value of the shape parameter, *σ*. Where the light-adapted membrane potential was >10 mV above the dark resting level the mean value of *t*_p_ decreased to 16.5±1.7 ms for green photoreceptors, 22.8±4.5 ms for blue, and 21.0±1.3 ms for UV (Fig. 2). The earlier response peak for green photoreceptors was significantly different from blue and UV (*p*<0.05; Kruskal-Wallis One Way Analysis of Variance on Ranks, followed by pairwise multiple comparisons with Dunn's method) but there was no difference in the mean *t*_p_ values between blue and UV photoreceptors. The mean values of the shape parameter, *σ*, for green, blue and UV photoreceptors were 0.29, 0.35, and 0.31, respectively (compared with 0.37, 0.36 and 0.35 for the corresponding values in the dark). Thus there was a trend for smaller *σ* values in green photoreceptors, although this only reached statistical significance when comparing green with blue photoreceptors in light-adapted conditions. The decreases in *t*_p_ values (and to a lesser extent *σ*) lead to overall shorter response durations in green photoreceptors compared with blue and UV. The response half-width, *Δt* , is approximated to an accuracy of 1% by the equation *Δt* = 2.35*σ t*_p_ [7], which gives response durations of 11.4, 18.6 and 15.2 ms for green blue and UV photoreceptors, respectively. These values are slightly greater than the comparable figures for *B. terrestris*, but show the same pattern of faster impulse responses in green photoreceptors. Although the mean and standard deviation for the impulse response data shown in Fig. 2 may suggest some degree of overlap between spectral classes, this may have been due to individual differences between bees. When we ranked photoreceptors separately from each of the three bees used in this study, according to either *t*_p_ or *Δt* , we found no overlap between green and blue or UV. In other words, in each bee, the slowest impulse responses of green photoreceptors were faster than the fastest impulse responses of both blue and UV photoreceptors.

**Figure 3:**
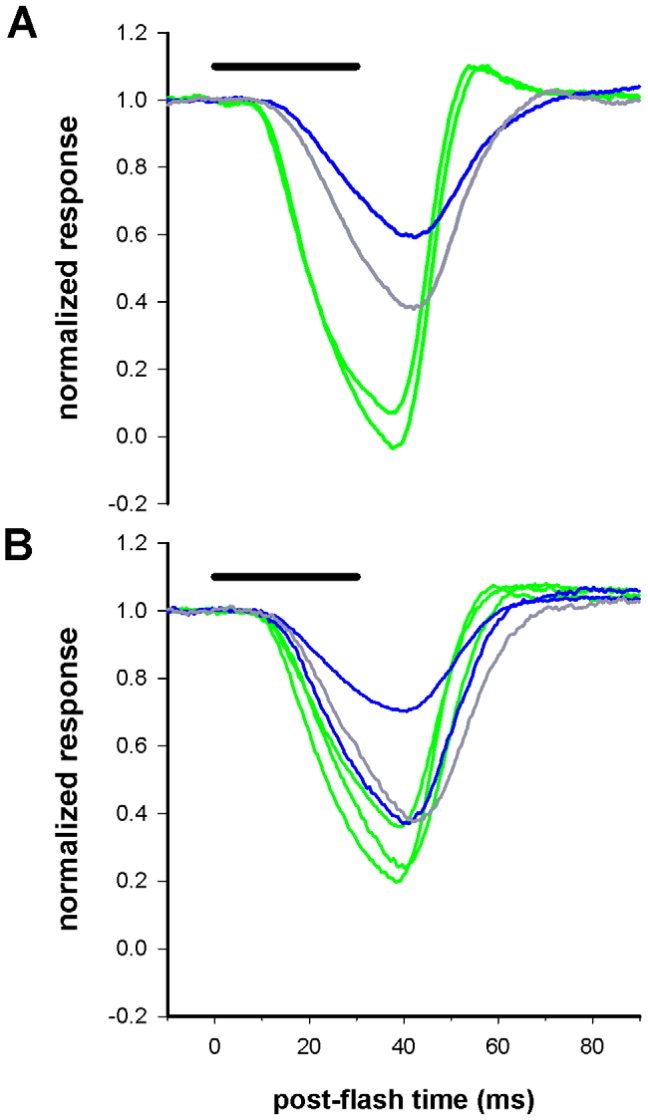
Responses to negative contrast steps compared in all three photoreceptor classes from two different bees. A. Superimposed averaged responses of four photoreceptors from the same bee. The adapting light was switched off for 30 (duration indicated by horizontal bar above traces) and the responses recorded (average of 20–30 sweeps in each case) in two green, one blue and one UV photoreceptor (spectral class denoted by green, blue and grey traces, respectively). Responses are normalized so that 0 represents the resting potential in the dark and 1 the mean depolarization of the membrane potential during light adaptation. B. Similar recordings from three green, two blue and one UV photoreceptor from a different bee. All other details as in *A*.

**Figure 4:**
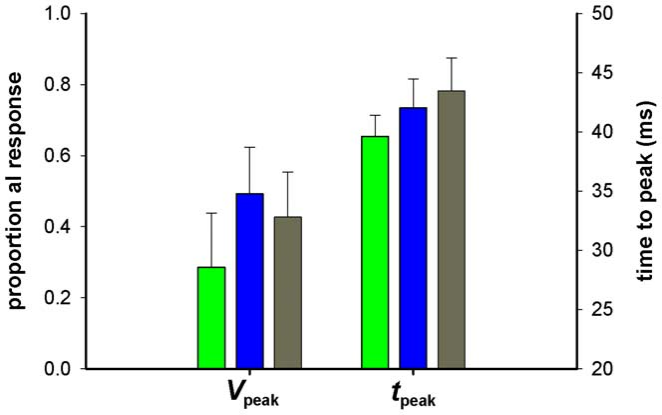
Mean parameters of light-off responses compared in all three spectral classes of photoreceptor. Left y-axis: light-adapted membrane potential ( = 1.0) normalized with respect to the dark resting potential (0). Mean light-adapted membrane potentials were 21.4±2.8 (green photoreceptors), 23.7±3.8 (blue), 24.4±3.2 mV (UV). *V*_peak_ plots the peak of the negative (repolarizing) response during a 30 ms pulse of darkness for green (green bars) blue (blue bars) and UV (grey bars) photoreceptors. *T*_peak_ is the latency to the peak negative response (right y-axis), measured from onset of dark pulse.

The faster impulse responses of green photoreceptors in *B. impatiens* were also correlated with a faster membrane potential repolarization following negative contrast steps, as in *B. terrestris*. Following light adaptation to a steady state membrane potential, depolarized from rest by 17–28 mV (21.4±2.8, 23.7±3.8, 24.4±3.2 mV for green, blue and UV, respectively) cells were tested with negative contrast steps of 30 ms duration (generated by setting the LED voltage to 0 for the duration of the step). We measured the response peak as the point of maximum repolarization, normalized on a scale where 1 corresponds to the steady state light-adapted membrane potential, and 0 to the resting potential in the dark (Fig. 3). Following onset of the negative contrast step photoreceptors began to repolarize within 7 to 14 ms, repolarizing maximally within 37–47 ms to a value of 0.7 to −0.03 relative to the light-adapted membrane potential (negative values here mean that the peak of the repolarizing response undershoots the dark resting potential). The off-responses generated in these experiments were larger, and somewhat faster, in green photoreceptors compared with blue or UV receptors (Fig. 4). There was no significant difference in response amplitude or time-to-peak between blue and UV photoreceptors, but green photoreceptors generated larger negative responses than blue (p<0.01), which peaked earlier than either blue (p<0.05) or UV (p<0.005). However, the increased amplitude of the peak negative (repolarizing) response in green compared with UV photoreceptors was not significant at the p = 0.05 level (p = 0.056).

**Figure 5:**
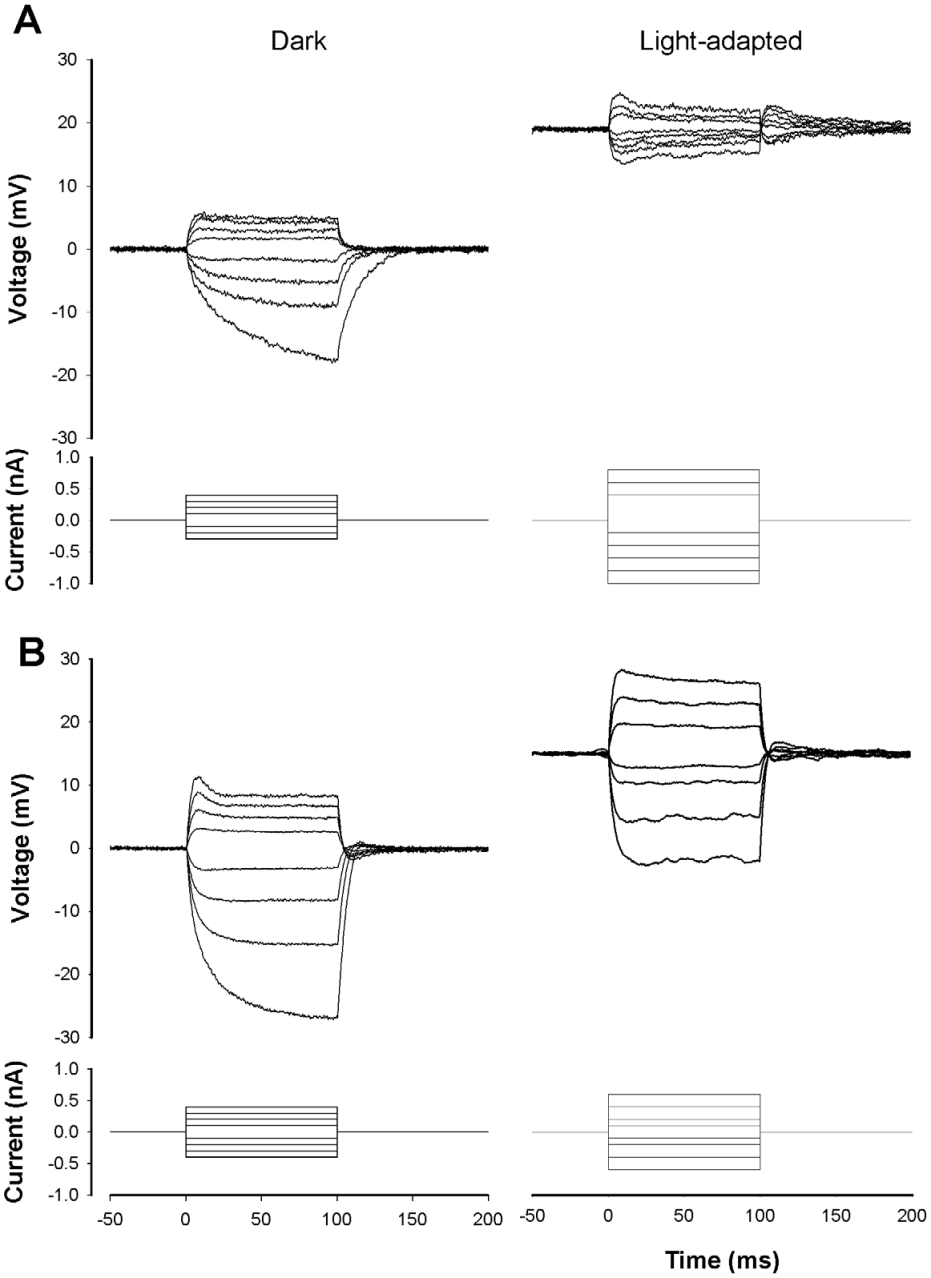
Dark and light adapted *I-V* relations compared in green and UV photoreceptor. A. Green photoreceptor. Superimposed membrane responses (upper traces) to square-wave current pulses (lower traces) injected in the dark (left) and during light adaptation. Resting potential in the dark is arbitrarily set to zero, and the vertical displacement of the light adapted resting level indicates the amplitude of the steady-state light-induced depolarization of the resting potential. B. As in *A*, for a recording from a UV photoreceptor.

**Figure 6:**
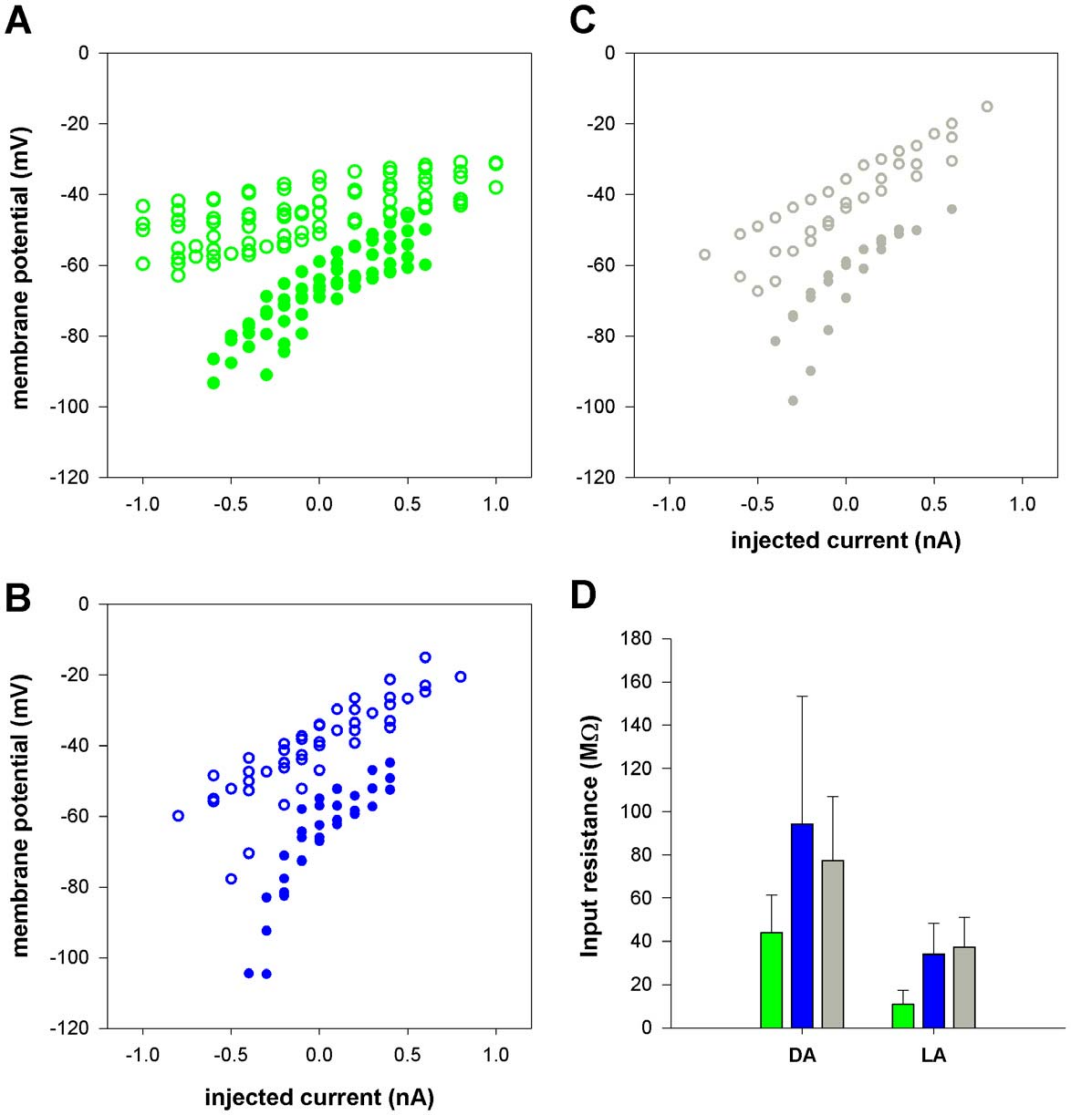
Relationship between membrane voltage and injected current for all three photoreceptor classes in the dark and during light adaptation. A–C. Membrane potential against injected current for green (A, n = 6 cells), blue (B, n = 4) and UV (C, n = 3) photoreceptors, in the dark (filled circles) and during light-adaptation (open circles). Induced changes in membrane potential were referred to the absolute resting potential, measured on withdrawal of the microelectrode (voltage values for 0 nA therefore correspond to a cell's resting potential, either in the dark or light-adapted). D. Estimated input resistance for all three photoreceptor spectral classes in dark (DA) and light-adapted (LA) conditions. Green, blue and grey bars denote green, blue and UV photoreceptors, respectively. Input resistance was estimated from the slope of the *V/I* function for negative current steps as illustrated in the data of Fig. 5; see text for further details.

The light-induced depolarization of insect photoreceptors is mediated by photo-activated conductances and then further shaped by activation of additional, voltage-dependent conductances [20]. To compare conductance changes induced by light-adaptation we examined voltage responses to current injection (current clamp) in all three classes of photoreceptor, in the dark and during light adaptation. Voltage responses to hyperpolarizing and depolarizing current pulses in the dark were asymmetrical in all photoreceptor spectral classes (Fig. 5), which is suggestive of voltage dependent conductances with activation thresholds near, or negative to, the resting potential. While hyperpolarizing responses to negative current pulses could often be fitted with a single exponential, approximating a simple RC charging, depolarizing responses were always more complex, suggesting activation of voltage-dependent conductances. All three photoreceptor classes showed evidence for activation of voltage-dependent conductances following depolarization from rest in the dark. In most photoreceptors the initial voltage response was a transient that rose rapidly to a peak, from which it decayed more slowly to a lower, plateau level of depolarization during the course of a 100 ms current pulse (Fig. 5). This type of response is typically due to the activation of a delayed rectifier potassium conductance. It was observed in all three spectral classes, but tended to be more prominent, and observed with smaller depolarizing responses in green photoreceptors. Plotting the voltage responses against injected current (Fig. 6) reveals the nonlinear *V/I* relationship, confirming the presence of voltage-gated conductances. All three photoreceptor spectral classes show rectification, evident as a decrease in slope of the *V/I* function at more positive membrane potentials, and it is also evident from the slope of the curves that the input resistance for green photoreceptors is lower than that for blue or UV photoreceptors at all levels of membrane potential.

For comparison of photoreceptor input resistances we fitted regression lines to the negative region of the V/I function for each cell. This yielded estimates of input resistance of 44.4±16.4 MΏ, 94.3±58.2 MΏ and 77.4±29.5 MΏ for green, blue and UV photoreceptors, respectively.

During light adaptation, input resistance decreased in all photoreceptor classes (Fig. 6), and the voltage changes induced by negative and positive current steps became more symmetrical, indicating a more linear slope of the *V/I* function. However, the waveform of the voltage responses was not indicative of simple RC charging; we frequently observed transient after de- and hyperpolarizations on termination of (respectively) hyperpolarizing and depolarizing responses. As can be seen from figure 6, the proportional decrease in input resistance was also greater in green photoreceptors (22%±7.9% of dark value; n = 7 cells) than in blue (41±19%; n = 5) or UV (49%±13%) photoreceptors.

## Discussion

Photoreceptor integration times are especially important in determining the resolving power of the eye during visual motion [21]. However, although photoreceptor spectral sensitivity information is available for many species [3], there are few studies of hymenopteran photoreceptors that provide electrophysiological data on response dynamics [4], [10], [22], [23]. Here we have measured temporal responses of all three spectral photoreceptor classes in the bumblebee, *B. impatiens*, extending our previous study in *B. terestris* [10]. In both species, light-adapted green photoreceptors (which are involved in fast achromatic vision) generate faster responses than blue or UV photoreceptors (which are involved in chromatic vision). Furthermore, we have shown in this study that the fall in input resistance during the light adapted state is greater in green photoreceptors than in blue or UV. There appears to be a species difference in absolute response speed: green photoreceptor integration times (*Δt*) are about 8 ms in *B. terrestris*, compared with about 11 ms in *B. impatiens*. Given that both these species are diurnal, generalist foragers, visiting a large range of variously coloured flower species and in operating in similar, temperate light climates, there would not appear to be any obvious explanation for the difference in terms of visual ecology. This difference notwithstanding, *B. impatiens* photoreceptors can be counted as relatively fast compared with a range of other diurnally active insects, where integration times range from about 5–20 ms (see table 3 in [10]). Mammalian cones have integration times of 20 ms upwards, and even larger values are found in rods and photoreceptors of nocturnal invertebrates [24]. It would be interesting to extend comparative studies to more closely related species with differing visual ecologies. For example, photoreceptor information capacity (which in turn depends on processing speed) appears to be sacrificed in a nocturnal bee compared with a closely-related diurnal species [23]. Since nocturnal colour vision has also been demonstrated in nocturnal insects, including bees [25] it would be very interesting to compare spectral and temporal properties across all photoreceptor classes in such species.

There is considerable behavioural evidence for parallel chromatic and achromatic channels in bees. For example, bees can perceive motion, depth, and form through depth cues using an achromatic visual channel, which is fed by inputs from green photoreceptors [11], [12], [26], [27]. Chromatic vision relies on comparison of signals from all three photoreceptor classes, and training experiments, where discriminations must be made by chromatic cues alone, reveal lower spatial resolution for the chromatic channel in both honeybees and bumblebees [12], [28], [29]. Since the temporal resolution for chromatic processing will be limited by the slowest photoreceptor inputs, it follows from the present results that the temporal resolution of the chromatic channel must also be lower. This was indeed found in behavioural measurements of chromatic and achromatic flicker fusion frequency in honeybees [30], [31]. Fast temporal processing is metabolically expensive, and it is increasingly recognized that metabolic cost is a major constraint on brain design [16]. For this reason one might expect that investment in such costs would be restricted to cases where fast temporal processing is essential, such as during visual motion. Where parallel channels for motion vision and chromatic vision are served by different photoreceptor classes, one might therefore expect the temporal response properties of these photoreceptors also to be different, reflecting the functional requirements of the different visual channels. Additionally, it could be that differences in photoreceptor responses reflect the requirements of downstream circuitry. In insects the second optic ganglion (the medulla) is thought to be the earliest stage in the visual system where chromatic information can be extracted. However, photoreceptors project to this stage by two different routes [32]. The long visual fibres (axons of blue and UV photoreceptors) project directly to this neuropil, wherease the short visual fibres (associated with green photoreceptors) terminate in the lamina (the first optic ganglion). Therefore signals from green photoreceptors are subject to synaptic processing in the lamina prior to the chromatic comparison stage in the medulla.

### Changes in photoreceptor input resistance during light adaptation

The increase in temporal resolution of the insect eye with light adaptation depends both on increased kinetics of the phototransduction cascade and the properties of the photoreceptor membrane [5], [6], [33]. The size and duration of responses to single photons are greatly reduced [33], [34] and at least three classes of voltage-dependent potassium channel contribute to an increase in the frequency bandwidth of the membrane [35], [36]. In flies, differences in the temporal resolving power of photoreceptors can result at least partly from tuning the membrane frequency response with voltage-gated potassium channels, which serve to reduce the membrane time constant and thus improve the frequency response in the depolarized, light-adapted state [5], [6], [33], [36], [37], [38]. The spectrum of voltage-activated membrane conductances differs between species with fast and slow photoreceptors, and also, within species, between (achromatic) R1-6 and (chromatic) R7-8 photoreceptors [8]. In the present study our measurements of input resistance also support a role for voltage-dependent membrane conductance in tuning photoreceptor frequency response. As would be expected, the tonic depolarization during light adaptation is associated with a decrease in input resistance, reflecting the opening of light-gated ion channels. However, this decrease is greatest in green photoreceptors, both in relative and absolute terms, despite the fact that the adapting intensities used generated very similar levels of tonic membrane depolarization in all three photoreceptor classes. Green photoreceptor input resistance in our light-adapted conditions was about 11 MΏ, representing a 75% decrease from the mean dark value of about 44 MΏ. Comparable changes in input resistance have also been reported in fly R1-6 (achromatic) photoreceptors [22], [39]. Furthermore, all though the *V/I* functions for all three photoreceptor classes in the dark showed rectification in the positive direction (Fig. 6A–C), indicative of the activation of voltage-dependent conductances, the slope in this region was further decreased during light adaptation in green, but not blue or UV photoreceptors. Full characterization of voltage-dependent conductances in worker bumblebee photoreceptors will require further work. Nevertheless, our results suggest that voltage-activated membrane conductances contribute to the greater temporal resolving power of bumblebee green photoreceptors.

### Functional considerations

Temporal and spatial resolution are inevitably linked since space translates into time during motion. However, much motion is self-generated, and assuming there is an internal signal correlated with the extent of self-generated movement, then the precise timing of photoreceptor signals potentially provides spatial information. It is also possible that visual motion may be of importance in extracting chromatic information in bees. The theoretical tradeoff between spatial and chromatic resolution is not the same in the compound eye and the vertebrate retina. If each ommatidium was equivalent to a pixel that could take on any chromaticity value, based on trichromatic sampling of the visual spectrum, then there would be no theoretical reason to expect difference in chromatic and achromatic spatial resolution in the compound eye. However, chromatic resolution is significantly lower than achromatic in both honeybees and bumblebees [28], [29]. It is now clear, moreover, that ommatidia are heterogeneous in terms of photoreceptor spectral sensitivity: all contain six green photoreceptors, but the remaining two principal photoreceptors may be blue, or UV sensitive, or both, giving rise to three distinct ommatidial spectral classes [40], [41]. Thus the chromaticity value of a point on the retinal image will depend not only on the spectral content of the signal at that point, but also which particular point it is. It could be that fine scanning of the retinal image improves chromatic resolution by correlating variation in the spectral signal with retinal location during scanning. We therefore conjecture that the relatively high speed of the early visual system in bumblebees may facilitate a form of active vision [42], both chromatic and achromatic.
